# Clinical predictors of seizure recurrence after the first post-ischemic stroke seizure

**DOI:** 10.1186/s12883-016-0729-6

**Published:** 2016-11-05

**Authors:** Hyeon Jin Kim, Kee Duk Park, Kyoung-Gyu Choi, Hyang Woon Lee

**Affiliations:** Departments of Neurology, Ewha Womans University School of Medicine and Ewha Medical Research Institute, Epilepsy and Sleep Center, Ewha Womans University Mokdong Hospital, 1071, Anyangcheon-ro, Yangcheon-gu, Seoul 158-710 South Korea

**Keywords:** Post-ischemic stroke seizure, Seizure recurrence, Epilepsy, Ischemic stroke

## Abstract

**Background:**

The number of patients suffering post-stroke seizure after ischemic stroke (PSSi) is quite considerable, especially because ischemic stroke is more prevalent than hemorrhage in the general population. This study aimed to determine the predicting factors for seizure recurrence in ischemic stroke survivors and develop a clinical scoring system for the prediction of risks for seizure recurrence after the first PSSi.

**Methods:**

We reviewed 3792 ischemic stroke patients from the Ewha Stroke Registry. A total of 124 (3.3 %) patients who experienced PSSi were recruited (mean follow-up for 44.4 months). Medical records concerning the etiology, functional disability, seizure onset latency from stroke, type of seizure, electroencephalography (EEG), and neuroimaging findings were statistically analyzed to derive a seizure recurrence risk scoring system.

**Results:**

Seizures recurred in 35.4 % (17/48) of early PSSi patients (≤1 week since stroke onset) and 48.7 % (37/76) of late PSSi (>1 week) patients. Atrial fibrillation, large sized, and cortical stroke lesion were more common in late onset PSSi compared to those in early onset PSSi (*p* < 0.05). Seizure recurrence tended to be more prevalent in early PSSi patients with male gender, atrial fibrillation or cortical stroke lesion, severe functional disability, and partial seizures. Seizure recurrence in late PSSi group was more common in patients of young age (≤65 years old), male gender, large lesion size, and partial seizure type. The validity of seizure recurrence risk score in the early PSSi group was better when evaluating based on gender, atrial fibrillation, cortical lesion, functional disability, and partial seizure type, with sensitivity of 70.6 % and specificity of 71.0 %.

**Conclusions:**

Our study characterized the high risk group for seizure recurrence in patients with the first PSSi. PSSi patients with high risk score of seizure recurrence had a greater chance of developing epilepsy later. Therefore, they should be considered for further treatment such as antiepileptic drug medication in clinical practice.

## Background

Stroke, one of the major causes of epilepsy, accounts for up to 11 % of epilepsy patients in western countries [1]. American Stroke Association has recently reported that about one in six (15.3 %) stroke survivors experiences seizures within about 3.4 years after suffering brain attack [2]. Disabling large cortical stroke lesions and hemorrhagic strokes are known to be associated with frequent acute symptomatic seizures [2–12]. In succession, stroke leads to a 23-fold increase in remote seizures and a 17-fold increase in epilepsy incidence rate after the acute phase [13]. However, these epidemiologic analyses have not provided clinical scales for the prediction of seizure recurrence nor treatment guidelines such as when to start anti-epileptic drug (AED) for those patients with high risk of seizure recurrence.

In fact, AEDs have been often prescribed for remote seizures due to higher likelihood of subsequent unprovoked events [11, 14]. However, AED prophylaxis is not generally considered to prevent development of future epilepsy regardless of etiology. The most debating point is whether AED is needed after the first acute symptomatic seizure. Up to date, there is no consensus on treatment option for patients with post-stroke seizure after ischemic stroke (PSSi) except individualization depending on each clinician's own criteria [15, 16], although there are some clinical evidence and guidelines on AED management for post-hemorrhagic stroke seizure (PSSh) patients [17, 18]. This is probably caused by the fact that seizures are less frequent complication after infarction (2–13.5 %) than hemorrhages (4.3–24 %) [2, 3, 5, 6, 11, 12]. However, the number of PSSi patients is pretty high because cerebral ischemia is much more prevalent than hemorrhage, especially in Korean population (76.1 % vs. 23.9 % respectively [19]), with increasingly ageing populations.

AED treatment has been generally recommended for PSSi patients after the second seizure event, but not for the first PSSi, except when status epilepticus occurs as seizure onset [20]. This recommendation is based on the investigated virtue that AEDs might reduce the occurrence of second seizure, although AEDs have no effect on the pathogenesis of disease or its late prognosis [21, 22]. The location of stroke lesions has been reported as an important clinical factor enduring predisposition to generate further seizures. In addition, several previous studies have verified that early PSSi is an independent risk factor for the development of late and recurrent seizures [9, 13].

Early seizure control is important because uncontrolled repetitive seizures may threaten patients not only through potential physical injuries, but also through deleterious effects on the brain with stroke lesion and degenerative changes considering the fact that patients are mainly the elderly. It is also plausible that recurrent peri-infarct depolarization might be harmful to already vulnerable tissues because the additional metabolic stress could cause further neuronal injuries [23, 24]. Although whether seizures are directly connected to worse stroke outcome remains controversial, seizures after stroke are known to be related to increased resource utilization and length of hospital stay as well as decreased survival at 30-day and 1-year time points [12, 25, 26].

The present study aimed to better define the clinical factors for predicting high risk of seizure recurrence after the first PSSi. Out study placed a special emphasis on the seizure recurrence predicting factors among ischemic stroke survivors, hopefully to develop practical AED treatment guideline. The proposed clinical scale for predicting seizure recurrence could provide clinicians with practical implications on ischemic stroke survivor management, such as identifying the population that might benefit from immediate AED intervention.

## Methods

### Patients

Among a total of 3792 consecutive patients admitted to Ewha Womans University Hospital between 2001 and 2012 due to cerebral infarction, 124 PSSi patients were included in this study. Data were obtained from medical records of their routine care for stroke and seizure at admission and outpatient department visits. The study endpoint of each individual was the last outpatient visit or seizure recurrence. Mean follow-up duration was 44.4 months from relevant ischemic stroke insult and 29.9 months from PSSi onset.

### Definitions

Acute stroke was defined as occurrence of acute neurological symptom of presumed vascular origin lasting 24 h or longer. It was confirmed by radiologic findings. Transient ischemic attack (TIA) was excluded. Primary hemorrhagic stroke and patients who were previously diagnosed with epilepsy or had probable epileptogenic lesions such as cerebral hemorrhage, traumatic brain injury, brain surgery, cortical dysplasia, brain tumor, and cerebral vascular malformation were also excluded. Radiologic evidence of clinically silent old lacunar infarctions, periventricular leukoaraiosis, and secondary hemorrhagic transformation of ischemia were not considered as exclusion criteria. In case of recurrent stroke, clinically significant and the most recent accident before the first seizure, regardless of lesion size and location, was considered as PSSi relevant stroke.

Seizure was diagnosed clinically, and distinguished as being partial or generalized, according to the 2010 International League Against Epilepsy (ILAE) criteria [27, 28]. Consequently, non-convulsive electroencephalographic (EEG) seizure was ruled out. Simple loss of consciousness or short-lasting episodes of mental confusion was not considered as sufficient for epileptic seizure diagnosis. Status epilepticus was defined as one continuous and unremitting seizure lasting for more than 5 min or recurrent seizures without restoration of consciousness for greater than 5 min [29]. Criteria for early and late PSSi were within 7 days or later from stroke onset [20, 30]. No distinction was made between acute symptomatic seizures and early PSSi. The recurred second seizure event was unprovoked and separated from the first one by more than 24 h according to the epilepsy definition [28].

### Clinical variables and indicators for seizure outcomes

To diagnose and determine the pathogenesis of stroke, all patients were investigated in a standardized fashion including neuroimaging modality (computed tomography [CT] or magnetic resonance imaging [MRI] with/without angiography). Duplex ultrasound and transesophageal or transthoracic echocardiography (TEE/TTE) were performed when necessary for further etiological classification (Trials of Org 10172 in Acute Stroke Treatment [TOAST] classification) [31].

Image was obtained within 24 h of PSSi relevant stroke onset. Infarct size was categorized as: small (≤15 × 15 mm and ≤ 5 slices), moderate (≤15 × 15 mm and > 5 slices or > 50 × 50 mm and ≤ 5 slices), and large (>50 × 50 mm and > 5 slices). Image follow-up was performed to rule out new epileptogenic lesions in the late PSSi group and patients with seizure recurrence. Functional disability was evaluated at discharge after stroke care based on the modified Rankin score (mRS) categorized as mild (mRS ≤ 1), moderate (2 ≤ mRS ≤ 3), or severe (mRS ≥ 4). Standard EEG of 20–30 min duration was performed within 24–72 h of PSSi onset and coded as normal, generalized slow, regional slow, or focal epileptiform discharge. Analysis was performed to determine whether EEG findings were correlated with relevant stroke lesion.

### Seizure recurrence risk scoring models

After compiling results from previous studies and our own results, we defined the following 10 clinical seizure recurrence predictors after the first PSSi: seizure onset age under 65 years old [3, 4, 12], male gender [10], atrial fibrillation, lesion size [9, 10, 12], cortex involvement [4, 5, 8–10, 12, 32], hemorrhagic transformation [5, 11, 32], functional disability consequence of stroke [3, 4, 7–9, 32, 33], status epilepticus [34], stroke lesion correlative EEG findings [33], and partial seizure. We derived and validated several scoring models based on the combination of these factors to predict seizure recurrence in early and late PSSi groups, respectively. Another two models from the Post-Stroke Epilepsy Risk Scale (PoSERS) studies [32] and the medical research council (MRC) Multicenter trial for Early Epilepsy and Single Seizures (MESS) [33] were also applied to our patients and compared to our own scales.

### Statistical analysis

All statistical data analyses were performed using SPSS ver. 21.0 (SPSS Inc., Chicago, IL, USA) and MedCalc programs (MedCalc Software Inc., Ostend, Belgium). Chi-square, Fisher’s exact test, and linear by linear association were used to compare categorical variables. Independent *t*-test was used for continuous variables to make univariable comparisons of clinical characteristics. Multivariable analysis was conducted using logistic regression model. Two-sided *p*-value of less than 0.05 was considered as statistically significant. Associations were evaluated by odd ratios (OR) with 95 % confidence intervals (CI). Receiver operating characteristic (ROC) curve analysis was conducted for performance measure of total scale and different subscales after item selection.

## Results

### Patient characteristics

**Fig. 1:**
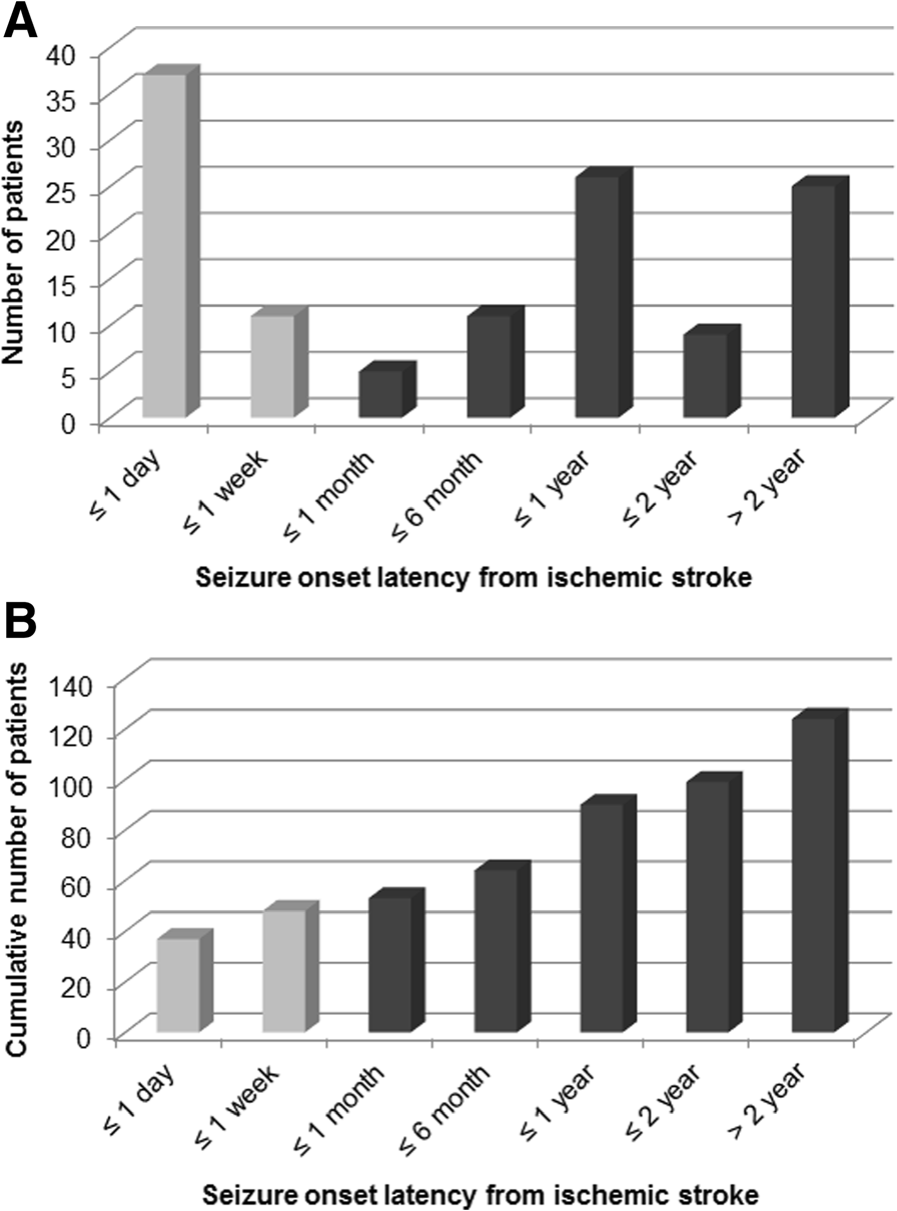
Latency of post-ischemic stroke seizure occurrence since cerebral infarction. **a** Distribution of seizure onset latency, and **b** cumulative number of patients. In the early onset group (*grey*), the first seizure occurred concurrently with stroke (within 24 h) in 77.1 % (37/48) of patients. In the late onset group (*black*), the latency exhibited a wide variation with a mean of 22.4 months. The peak period was at 6 months to 1 year after stroke

A total of 124 (3.3 %) PSSi occurred in 3792 ischemic stroke patients. There were 69 males and 55 females (1.3:1). The median age of patients with first seizure after ischemic stroke was 68 years old. Of the 124 patients, 48 (38.7 %) had early seizures. The remaining 76 (61.3 %) had late seizures. Among the 48 early PSSi patients, 77.1 % (37/48) of seizures occurred concurrently within 24 h after stroke onset (Fig. 1). Mean latency between stroke and late PSSi was 22.4 months (range, 9 days to more than 10 years), with peak time point of 6–12 months after stroke insult. Mean follow-up duration was 44.4 months after stroke and 29.9 months after the first seizure.

### Early vs. Late post-ischemic stroke seizures

The clinical characteristics of patients in the early and late PSSi groups are summarized in Table 1. No significant difference in seizure onset age, sex, or history of diabetes mellitus and hypertension was found between the two groups. However, atrial fibrillation was more prevalent in the late seizure group compared to that in the early PSSi group (36.8 % vs. 16.7 %, *p* = 0.016). The most common stroke etiology was large artery atherosclerosis (LAA) in both groups (54.2 % vs. 56.6 %). The second common etiology was small vessel occlusion (SVO) (18.8 %) in the early seizure group and cardioembolism (CE) (28.9 %) in the late seizure group. This was different from the statistics of Korean Clinical Research Center for Stroke which reported the etiology distribution was 36.1 % for LAA, 17.1 % for CE, and 25.4 % for SVO [19]. Large-sized lesions and cortical involvement were significantly more present in late PSSi (*p* = 0.001 and *p* = 0.002, respectively) compared to those in early PSSi. However, secondary hemorrhagic transformation frequencies of these lesions were not significantly different between the two groups. Vascular territory distribution of lesion was concentrated in the middle cerebral artery (MCA) territory in both groups (47.9 % vs. 69.7 %). Patients with mild functional disability were more common in early PSSi group than those in the late PSSi group (33.3 % vs 15.8 %). However, there was no statistical significance in vascular distribution or functional disability. Clinical seizure types (partial, generalized, or status epilepticus) were not significantly different between the two PSSi groups. The prevalence of lesion relevant EEG findings were 60.0 % (27/45) in early PSSi and 73.2 % (52/71) in late PSSi. EEG findings were not available for three early PSSi patients without seizure recurrence and five late PSSi patients (two of them had seizure recurrence). The seizure recurrence rate was 35.4 % (17/48) after early seizure and 48.7 % (37/76) after late seizure.

**Table 1:** Comparison of clinical characteristics related to early and late onset post-ischemic stroke seizures

Clinical characteristics		Total	Early onset	Late onset	*p*-value	OR (95 % CI)
		(*N* = 124)	(*N* = 48)	(*N* = 76)		
Age (years)	Median	68.0	68.0	69.0	0.670	
	Interquartile range	57.0–75.0	55.5–75.8	59.0–74.5		
Gender	Male	69	29 (60.4 %)	40 (52.6 %)		
	Female	55	19 (39.6 %)	36 (47.4 %)	0.395	1.374 (0.660–2.859)
Diabetes mellitus		44	20 (41.7 %)	24 (31.6 %)	0.253	0.646 (0.305–1.369)
Hypertension		89	33 (68.8 %)	56 (73.7 %)	0.552	1.273 (0.574–2.820)
Atrial fibrillation		36	8 (16.7 %)	28 (36.8 %)	0.016*	2.917 (1.197–7.108)
TOAST classification	LAA	69	26 (54.2 %)	43 (56.6 %)		
	CE	26	4 (10.4 %)	22 (28.9 %)		
	SVO	16	9 (18.8 %)	7 (9.2 %)		
	Other determined	2	1 (2.1 %)	1 (1.3 %)		
	Undetermined	10	7 (14.6 %)	3 (3.9 %)		
Lesion size	Small	10	7 (14.6 %)	3 (3.9 %)		
	Moderate	31	17 (35.4 %)	14 (18.4 %)		
	Large	83	24 (50.0 %)	59 (77.6 %)	0.001**	
Cortical involvement		106	35 (72.9 %)	71 (93.4 %)	0.002**	5.274 (1.742–15.972)
Hemorrhagic transformation		28	8 (16.7 %)	20 (26.3 %)	0.211	1.786 (0.715–4.458)
Vascular territory	ACA	9	3 (6.3 %)	6 (7.9 %)		
	MCA	76	23 (47.9 %)	53 (69.7 %)		
	ICA	13	6 (12.5 %)	7 (9.2 %)		
	Lacune	10	7 (14.6 %)	3 (3.9 %)		
	Watershed	6	4 (8.3 %)	2 (2.6 %)		
	PCA	7	3 (6.3 %)	4 (5.3 %)		
	Venous	2	1 (2.1 %)	1 (1.3 %)		
	Brainstem	1	1 (2.1 %)	0		
Functional disability	Mild	28	16 (33.3 %)	12 (15.8 %)		
	Moderate	35	6 (12.5 %)	29 (38.2 %)		
	Severe	61	26 (54.2 %)	35 (46.1 %)	0.527	
Status epilepticus		33	13 (27.1 %)	20 (26.3 %)	0.925	0.962 (0.425–2.175)
EEG findings	Normal	20	9 (18.8 %)	11 (14.5 %)		
(*n* = 116)	Generalized slow	17	9 (18.8 %)	8 (10.5 %)		
	Regional slow	59	19 (39.6 %)	40 (52.6 %)		
	Epileptiform discharge	20	8 (16.7 %)	12 (15.8 %)	0.136^a^	1.825 (0.824–4.040)
Clinical seizure type	Generalized	68	23 (47.9 %)	45 (59.2 %)		
	Partial	56	25 (52.1 %)	31 (40.8 %)	0.218	0.634 (0.306–1.312)
Seizure recurrence		54	17 (35.4 %)	37 (48.7 %)	0.147	1.730 (0.823–3.637)

*OR* odds ratio, *CI* confidence interval, *TOAST* Trials of Org 10172 in Acute Stroke Treatment, *LAA* Large artery atherosclerosis, *CE* Cardioembolism, *SVO* Small vessel occlusion, *ACA* anterior cerebral artery, *MCA* middle cerebral artery, *ICA* internal cerebral artery, *PCA* posterior cerebral artery, *EEG* electroencephalography

Statistical significance with *p* < 0.05* and *p* < 0.01**

^a^ Variables were substituted whether EEG findings were correlated with the relevant stroke lesion to evaluate the *p*-value

Each patient's AED treatment regimen (Table 2) was decided by the treating neurologist. In the early seizure group, 14.6 % (7/48) patients were not prescribed AED after their first seizure. However, AED was started later in three of these patients due to second unprovoked seizure. Among early PSSi patients administered with AEDs, 11 patients had no subsequent seizure even after AED discontinuation whereas 11 patients had the second seizure despite of continuous AED taking. In the late PSSi group, 9.2 % (7/76) patients were not prescribed AED after the first seizure, and all of them had the second unprovoked seizure. Among the 22 AED discontinued patients in the late PSSi group, 14 developed second seizure later while the remaining 8 patients did not have second seizure.

**Table 2:** Antiepileptic drug regimen of post-ischemic stroke seizures patients

	Early onset PSSi (*N* = 48)			Late onset PSSi (*N* = 76)		
AED prescription	None	Discontinued	Continues	None	Discontinued	Continues
	(*N* = 7)	(*N* = 14)	(*N* = 27)	(*N* = 7)	(*N* = 22)	(*N* = 47)
Not recurred	4	11	16	0	8	31
Recurred	3	3	11	7	14	16

*AED* antiepileptic drug, *PSSi* post-stroke seizure after ischemic stroke/post-ischemic stroke seizure

`

### Single vs. recurrent seizures among early post-ischemic stroke seizure patients

Seizure recurrence related clinical factors in the early PSSi group were investigated, and results are shown in Table 3. Males were more common in the recurrent seizure group compared to females (70.6 % vs. 54.8 %, *p* = 0.033 in multivariable analysis). History of diabetes mellitus and hypertension were more common in the single seizure group compared to those in the recurrent seizure group. Atrial fibrillation was more prevalent in recurrent seizure group compared to that in the single seizure group (29.4 % vs. 9.7 %). Cortical involvement was more common in the recurrent seizure group (82.4 % vs. 67.7 %), whereas hemorrhagic transformation was more common in the single seizure group (19.4 % vs. 11.8 %). Severely disabled patients were more prevalent in patient with later seizure recurrence (64.7 % vs. 48.4 %). Partial seizure type was more common in the recurrent seizure group (54.7 % vs. 45.2 %).

**Table 3:** Clinical characteristics related to seizure recurrence after early onset post-ischemic stroke seizure

Clinical characteristics	Not recurred	Recurred	Univariable analysis		Multivariable analysis	
	(*N* = 31)	(*N* = 17)	*p*-value	OR (95 % CI)	*p*-value	OR (95 % CI)
Age (years old)	65.7 ± 14.9	66.1 ± 12.6	0.941		0.882^a^	0.880 (0.161–4.799)
Male gender	17 (54.8 %)	12 (70.6 %)	0.286	1.976 (0.560–6.971)	0.033*	11.822 (1.222–114.347)
Diabetes mellitus	16 (51.6 %)	4 (23.5 %)	0.059	0.288 (0.077–1.084)	0.347	0.346 (0.038–3.167)
Hypertension	23 (74.2 %)	10 (58.8 %)	0.272	0.497 (0.141–1.747)	0.355	0.353 (0.039–3.206)
Atrial fibrillation	3 (9.7 %)	5 (29.4 %)	0.112	3.889 (0.799–18.938)	0.106	10.451 (0.609–179.306)
Lesion size						
Small	4 (12.9 %)	3 (17.6 %)			0.360	
Moderate	13 (41.9 %)	4 (23.5 %)			0.576	2.779 (0.077–99.920)
Large	14 (45.2 %)	10 (58.8 %)	0.468		0.440	0.412 (0.043–3.910)
Cortical involvement	21 (67.7 %)	14 (82.4 %)	0.330	2.222 (0.518–9.537)	0.163	6.406 (0.471–87.078)
Hemorrhagic transformation	6 (19.4 %)	2 (11.8 %)	0.694	0.556 (0.099–3.114)	0.197	0.155 (0.009–2.632)
Functional disability						
Mild	12 (38.7 %)	4 (23.5 %)			0.402	
Moderate	4 (12.9 %)	2 (11.8 %)			0.564	0.386 (0.015–9.788)
Severe	15 (48.4 %)	11 (64.7 %)	0.532		0.293	3.517 (0.349–32.620)
Status epilepticus	8 (25.8 %)	5 (29.4 %)	1.000	1.198 (0.321–4.473)	0.941	1.077 (0.151–7.673)
Relevant EEG findings (*N* = 45)	17 (54.8 %)	10 (58.8 %)	0.900	0.924 (0.271–3.156)	0.951	1.067 (0.134–8.512)
Partial seizure type	14 (45.2 %)	11 (64.7 %)	0.195	2.226 (0.657–7.545)	0.159	4.619 (0.546–38.873)

*OR* odds ratio, *CI* confidence interval, *EEG* electroencephalography

Statistical significance with *p* < 0.05*

^a^ Variables were substituted whether patients were younger than 65 years old

### Single vs. recurrent seizures among late post-ischemic stroke seizure patients

Seizure recurrence related clinical factors in the late PSSi group are summarized in Table 4. Young age and male gender were significantly correlated with higher seizure recurrence rate in both univariable and multivariable analyses (*p* = 0.050 and *p* = 0.027, respectively). Among lesion related radiologic findings, large lesion size was slightly more prevalent in the recurrent seizure group compared to that in the single seizure group (81.1 % vs. 74.4 %). Patients with later seizure recurrence exhibited more tendency to have partial seizure (45.9 % vs. 35.9 %) but less tendency to have status epilepticus (16.2 % vs. 35.9 %) and lesion relevant EEG abnormalities (59.5 % vs. 76.9 %) than those with single seizure.

**Table 4:** Clinical characteristics related to seizure recurrence after late onset of post-ischemic stroke seizure

Clinical characteristics	Not recurred	Recurred	Univariable analysis		Multivariable analysis	
	(*N* = 39)	(*N* = 37)	*p*-value	OR (95 % CI)	*p*-value	OR (95 % CI)
Age (years old)	70.7 ± 9.1	62.7 ± 11.9	0.001**		0.050*^a^	3.746 (1.000–14.040)
Male gender	14 (35.9 %)	26 (70.3 %)	0.003**	4.221 (1.613–11.043)	0.027*	4.552 (1.183–17.511)
Diabetes mellitus	14 (35.9 %)	10 (27.0 %)	0.406	0.661 (0.249–1.757)	0.460	0.578 (0.135–2.476)
Hypertension	31 (79.5 %)	25 (67.6 %)	0.238	0.538 (0.190–1.518)	0.451	0.566 (0.129–2.488)
Atrial fibrillation	16 (41.0 %)	12 (32.4 %)	0.438	0.690 (0.270–1.764)	0.521	0.647 (0.171–2.443)
Lesion size						
Small	2 (5.1 %)	1 (2.7 %)			0.581	
Moderate	8 (20.5 %)	6 (16.2 %)			0.999	-
Large	29 (74.4 %)	30 (81.1 %)	0.520		0.298	0.329 (0.041–2.667)
Cortical involvement	37 (94.9 %)	34 (91.9 %)	0.671	0.613 (0.096–3.891)	0.999	-
Hemorrhagic transformation	7 (17.9 %)	13 (35.1 %)	0.089	2.476 (0.858–7.150)	0.688	1.370 (0.296–6.347)
Functional disability						
Mild	5 (12.8 %)	7 (18.9 %)			0.802	
Moderate	15 (38.5 %)	14 (37.8 %)			0.854	1.216 (0.151–9.796)
Severe	19 (48.7 %)	16 (43.2 %)	0.490		0.558	1.833 (0.205–16.406)
Status epilepticus	14 (35.9 %)	6 (16.2 %)	0.051*	0.346 (0.116–1.030)	0.057	0.208 (0.041–1.048)
Relevant EEG findings (*N* = 71)	30 (76.9 %)	22 (59.5 %)	0.051*	0.338 (0.111–1.030)	0.118	0.294 (0.063–1.367)
Partial seizure type	14 (35.9 %)	17 (45.9 %)	0.373	1.518 (0.605–3.808)	0.237	2.227 (0.590–8.404)

*OR* odd ratio, *CI* confidence interval, *EEG* electroencephalography

Statistical significance with *p* < 0.05* and *p* < 0.01**

^a^ Variables were substituted whether patients were younger than 65 years

### Scoring models for predicting seizure recurrence

**Fig. 2:**
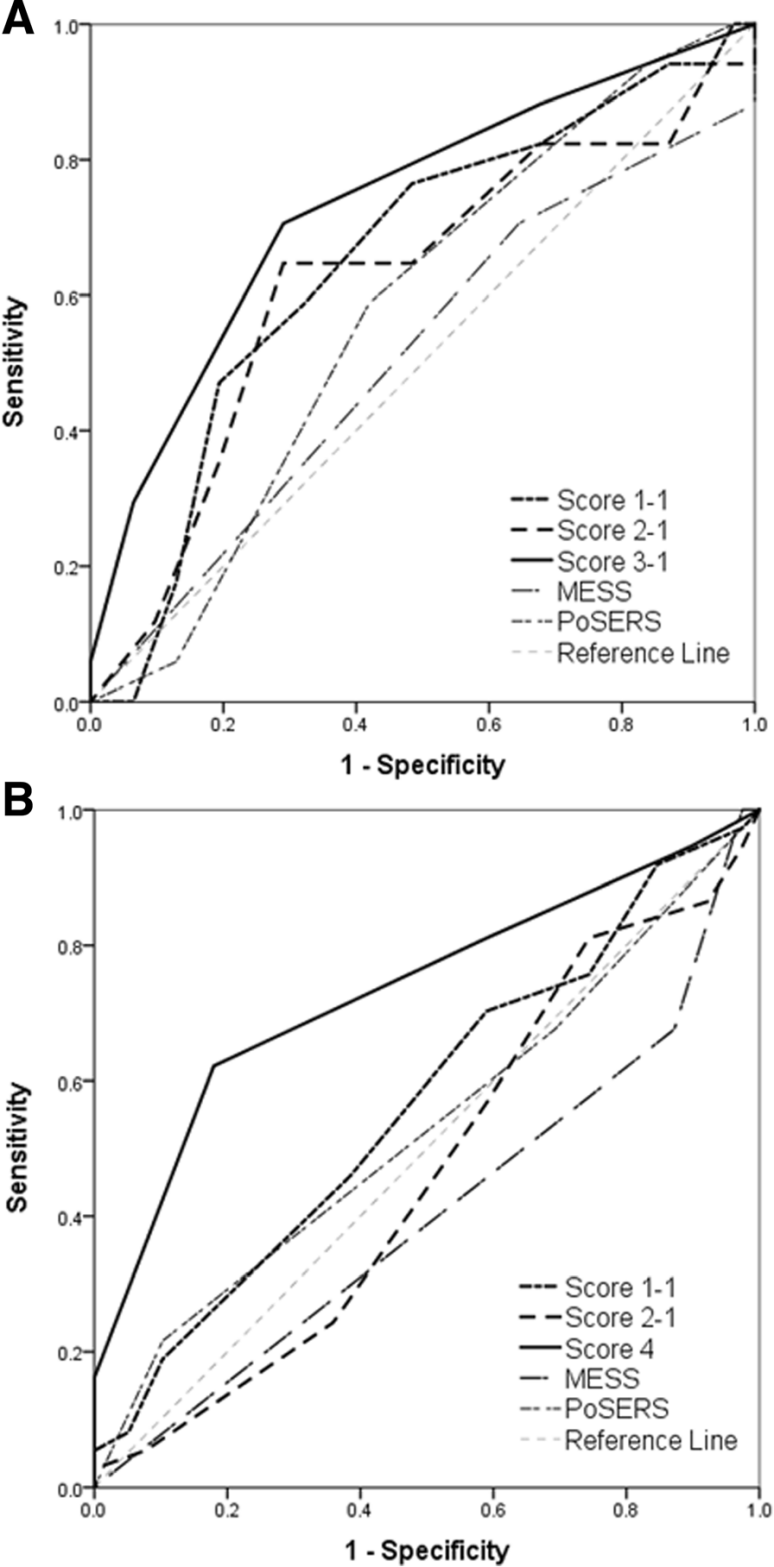
Receiver operating characteristic curves for the seizure recurrence risk score models. **a** Validation in the early onset post-ischemic stroke seizure (PSSi) group, and **b** validation in the late onset PSSi group. Score 1-1 included all 10 items, whereas score 2-1 excluded age, sex, and atrial fibrillation factors. Score 3-1 (male sex, atrial fibrillation, cortical involvement, functional disability, and partial seizure) in early onset and score 4 (younger age at seizure onset, male sex, large stroke lesion, and partial seizure) in late onset patients (solid line) were composed of more prevalent items (*p* < 0.3) in each recurrent group that showed the largest area under the curve (AUC) among each group’s suggested scores. Predictive scores from MRC Multicenter trial for Early Epilepsy and Single Seizures (MESS) and Post-Stroke Epilepsy Risk Scale (PoSERS) study were applied to compare our patients. However, they demonstrated little efficacy for detecting or discriminating seizure recurrence in patients with early and late onset PSSi

The validities of scoring models for seizure recurrence risk measure are summarized in Table 5 and Fig. 2. Score 1 included all the above-mentioned 10 clinical predictors, whereas score 2 was concentrated on lesion related factors after excluding demographic (sex and age) and comorbid factors (atrial fibrillation). Score 3 and score 4 included items more prevalent in seizure recurrence groups (*p* < 0.3 in multivariable analysis) of early and late PSSi, respectively. These models exhibited the best performance with highest area under the curve (AUC) of 0.735 in early PSSi and 0.734 in late PSSi. By substituting disability categorical variables with raw mRS data (Score O − 2 version), each scale was found to have higher specificity but slightly lower sensitivity than the previous version (Score O − 1 version). Overall, the validity of seizure recurrence risk score in the early PSSi group was better than other models when evaluating based on gender, atrial fibrillation, cortical lesion, functional disability, and partial seizure type, with sensitivity of 70.6 % and specificity of 71.0 %. In late PSSi group, score model based on age, gender, lesion size, and partial seizure type was exhibited best performance, with sensitivity of 62.2 % and specificity of 81.1 %. Two prognostic score models (MESS and PoSERS) reported in previous studies were also used to compare our study subjects [32, 33]. Both MESS and PoSERS demonstrated better specificities than our own scales for late PSSi. However, their sensitivities were much lower than our scales.

**Table 5:** Validity tests for post-ischemic stroke seizure recurrence risk score models

	Version	Items^a^	AUC	95 % CI	Criteria	Sensitivity	Specificity	+ LR	- LR
Early	Score 1-1	Y, M, A, B, C, H, D, E, F, P	0.653	0.502–0.784	6	58.8 %	67.7 %	1.82	0.61
PSSi	Score 1-2	Y, M, A, B, C, H, mRS, E, F, P	0.650	0499–0.782	9	52.9 %	80.7 %	2.74	0.58
	Score 2-1	B, C, H, D, E, F, P	0.621	0.470–0.757	5	64.7 %	71.0 %	2.23	0.50
	Score 2-2	B, C, H, mRS, E, F, P	0.622	0.471–0.758	8	52.9 %	80.7 %	2.74	0.58
	Score 3-1	M, A, C, D, P	0.735	0.588–0.852	3	70.6 %	71.0 %	2.43	0.41
	Score 3-2	M, A, C, mRS, P	0.676	0.525–0.803	6	47.1 %	80.7 %	2.43	0.66
	MESS	mRS ≥ 1, abnormal EEG	0.509	0.361–0.657	1	70.6 %	35.5 %	1.09	0.83
	PoSERS	Supratentorial (X 2), C, H, mRS ≥ 3	0.576	0.425–0.717	3	58.8 %	58.1 %	1.40	0.71
Late	Score 1-1	Y, M, A, B, C, H, D, E, F, P	0.566	0.447–0.679	7	46.0 %	61.5 %	1.19	0.88
PSSi	Score 1-2	Y, M, A, B, C, H, mRS, E, F, P	0.558	0.439–0.671	9	40.5 %	69.2 %	1.32	0.86
	Score 2-1	B, C, H, D, E, F, P	0.534	0.416–0.649	5	43.2 %	59.0 %	1.05	0.96
	Score 2-2	B, C, H, mRS, E, F, P	0.526	0.408–0.642	7	54.1 %	59.0 %	1.32	0.78
	Score 4	Y, M, B, P	0.734	0.620–0.829	3	62.2 %	81.1 %	3.46	0.46
	MESS	mRS ≥ 1, abnormal EEG	0.594	0.475–0.705	1	32.4 %	87.2 %	2.53	0.78
	PoSERS	Supratentorial (X 2), C, H, mRS ≥ 3	0.532	0.414–0.647	4	21.6 %	89.7 %	2.11	0.87

*AUC* area under the curve, *CI* confidence interval, *+LR* positive likely-hood ratio, *-LR* negative likely-hood ratio, *mRS* modified Rankin Score, *MESS* MRC Multicenter trial for Early Epilepsy and Single Seizure, *PoSERS* Post-Stroke Epilepsy Risk Scale, *EEG* electroencephalography

^a^ Y, younger age - below 65 years old (1); M, male (1); A, atrial fibrillation (1); B, bigger lesion size - small (0), moderate (1), large (2); C, cortical involvement (1); H, hemorrhagic transformation (1); D, functional disability - mild (0), moderate (1), severe (2); E, status epilepticus (1); F, relevant focal EEG finding (1); P, partial seizure (1)

## Discussion

PSSi is increasingly encountered in clinical practice as population ages. A tool to identify patients with high risk of recurrent unprovoked seizures would benefit such patients. However, such tool is currently unavailable because previous studies mainly reported the PSS incidence related factors rather than seizure recurrence predicting factors without distinction of stroke etiology. This could be an important process to identify epilepsy patients in accordance with the newly proposed ILAE definition in 2014 as being after single seizure with enduring predisposition to recurrent seizures at a chance of greater than 60 % [28, 35].

The main findings of our study are as follows: (1) the overall prevalence of PSSi among ischemic stroke survivors was 3.3 % (124/3792); (2) seizure recurred patients among early PSSi (35.4 %, 17/48) exhibited similar tendency to the late PSSi group rather than the general early PSSi group in some clinical characteristics - atrial fibrillation, cortical stroke lesion - in addition to the prevalence of male gender, severe functional disability, and partial seizure type; (3) seizure recurrence in the late PSSi group (48.7 %, 37/76) was more common in patients of younger age (≤65 years old), male gender, large lesion size, and partial seizure type; (4) the validity of seizure recurrence risk score with these clinical variables was improved with sensitivity of 70.6 % and specificity of 71.0 % for the early PSSi group, and with sensitivity of 62.2 % and specificity of 81.1 % for the late PSSi group, respectively.

It has been reported that seizure onset latency from antecedent is one of the major AED prescription criteria of acquired epilepsy based on the investigated risk difference of subsequent event up to 80 % [9, 11, 14]. Therefore, early and late PSSi groups were compared in this study. Different prognostic factors including seizure recurrence rate are regarded as a reflection of underlying pathomechanism associated with the enduring predisposition. Early seizures are thought to result from acute disruption of brain integrity, metabolic homeostasis, and excitatory glutamate release, leading to secondary neuronal injury and electrically irritable tissue. In contrast, late seizures are related to neuronal circuit reorganization by aberrant gliosis and development of hyperexcitable cicatrix, eventually resulting in spontaneous seizures [23, 36]. We hypothesized that clinical characteristics of PSSi patients might reflect the epileptogenic predisposition of post-stroke brain. Our results suggested that different epilepsy pathogenesis might be involved in early and late PSSi because the adjusted ORs of recurrence related factors did not match each other.

In our study, significantly different predisposing factors between early and late PSSi groups were: atrial fibrillation history, large lesion size, and cortical involvement of lesion. Seizure recurrence rate between the two groups did not reach statistical significance possibly due to the relatively lower recurrence rate in the late onset PSSi group compared with other large center studies (12.4–57.1 %) [3, 4, 6, 8]. The ascertained item lists not only contained plausible factors for acquired localization-related epilepsy, but also contained implausible demographic factors (age, sex) and comorbid factors (e.g. atrial fibrillation). Although establishing causality based on a statistical association is hazardous, excluding the latter factors from the suggested score system failed to enhance the sensitivity or specificity of seizure recurrence risk prediction. Considering lower volume of cortical gray matter and decreased excitability due to degenerative changes, it is easy to assume that younger patients might develop seizures more often [37]. As for gender, hormone effect especially progesterone can account for difference in promoting and enhancing repair after traumatic brain injury and stroke [38]. The association between seizure and cardioembolism is often mentioned in published studies [8, 39]. However, such association remains controversial due to various study designs and diagnostic testing. Another considering point is confounders and interaction between predictors, such as age with seizure manifestation, survival rate, atrial fibrillation with cortical involvement, and so on.

Among those relatively more plausible epileptogenic lesion-related factors, cortical location was consistently found to be an associated factor for the determination of the probability of seizure. Thus, cortical location can be an important clinical factor to decide for AED treatment in clinical practice. The interaction effect between predictors, especially with lesion size or severe functional disability, should be further evaluated with large number of patients to validate and enhance the specificity of suggested scale. Other factors exhibited discrepancies between our results and those from previous studies, possibly due to our specific focus on seizure recurrence and etiology limitation to ischemic stroke. Extravasation and irritation of blood metabolism product might have epileptogenic role in acute phase of stroke [40]. However, our study result suggest a low correlation between hemorrhagic transformation of ischemia and seizure recurrence. Clinical partial seizure type exhibited significant positive trend of seizure recurrence in our study, whereas EEG finding was insignificant. EEG finding of post-stroke brain especially in acute phase has relatively low diagnostic value for seizure [41], as it reflects acute dysfunction of post-stroke brain rather than epileptogenic activity. The correlation between status epilepticus and seizure recurrence was strong in patients with early PSSi. This might be associated with severe and large stroke lesion in those patients presenting status epilepticus as early seizures [34].

Although clinicians always deliberately weigh the pros and cons of AED administration in each individual, inconsistent rules might result in inappropriate treatment. In our subjects, there were untreated AED required for 3 early and 7 late PSSi patients and overestimated AED taken by 11 early and 8 late PSSi patients. Consistent application of evidenced guideline based on seizure recurrence risk measure with high specificity could decrease gratuitous side effects and interactions of AED with many other drugs taken by disabled elderly patient. The higher predictive power of our own score than scores of other previous studies without distinction of stroke etiology indicates the necessity of distinct score exclusively for ischemic stroke etiology.

Our study have several limitations. First, we reviewed the patients’ medical records retrospectively, so seizure events could have been missed or underdiagnosed in some patients. Second, the observational nature of our study nature is prone to uncontrolled AED prescription, variable follow up duration, or bias of unmeasured factors. Lastly, the small number of patients in this study might have limited to derive adjusted ORs with multiple predictor variables. However, we tested the distribution of all continuous variables and confirmed that they are normally distributed. Although we selected clinical variables using a modest statistical guideline of *p* < 0.3, we found that the validity of our scoring systems was improved with relatively good sensitivity and specificity. This kind of clinical scoring system for seizure recurrence in PSSi patients could be useful with further validation in the future large scale prospective study.

## Conclusion

Currently, AED treatment protocol for PSSi patients, especially those with early seizure onset, is dependent on variable and indefinite reference of each clinician. The proposed score confined to ischemic stroke survivors may help clinicians not only in AED treatment decision, but also in patient selection for further prospective randomized trials, preferably after supplement of case number and external validation to establish generalizability.

## Abbreviations

AED: Antiepileptic drug
AUC: Area under the curve
CE: Cardioembolism
CI: Confidence interval
CT: Computed tomography
EEG: Electroencephalography
ILAE: International League Against Epilepsy
LAA: Large artery atherosclerosis
MCA: Middle cerebral artery
MESS: Multicenter trial for Early Epilepsy and Single Seizures
MRC: Medical research council
MRI: Magnetic resonance imaging
mRS: Modified Rankin score
OR: Odd ratio
PoSERS: Post-Stroke Epilepsy Risk Scale
Pssh: Post-stroke seizure after hemorrhagic stroke/post-hemorrhagic stroke seizure
PSSi: Post-stroke seizure after ischemic stroke/post-ischemic stroke seizure
ROC: Receiver operating characteristic
SVO: Small vessel occlusion
TEE: Transesophageal echocardiography
TIA: Transient ischemic attack
TOAST: Trials of Org 10172 in Acute Stroke Treatment
TTE: Transthoracic echocardiography

## References

[1] Hauser WA, Annegers JF, Kurland LT (1993). Incidence of epilepsy and unprovoked seizures in Rochester, Minnesota: 1935–1984. Epilepsia.

[2] Merkler AE, Dunn LE, Lerario MP, Morris NA, Gialdini G, Kummer BR, Lahiri S, Navi BB, Kamel H (2016). The Long-Term Risk of Seizures After Stroke. Stroke.

[3] Conrad J, Pawlowski M, Dogan M, Kovac S, Ritter MA, Evers S (2013). Seizures after cerebrovascular events: risk factors and clinical features. Seizure.

[4] Graham NS, Crichton S, Koutroumanidis M, Wolfe CD, Rudd AG (2013). Incidence and associations of poststroke epilepsy: the prospective South London Stroke Register. Stroke.

[5] Beghi E, D'Alessandro R, Beretta S, Consoli D, Crespi V, Delaj L, Gandolfo C, Greco G, La Neve A, Manfredi M (2011). Incidence and predictors of acute symptomatic seizures after stroke. Neurology.

[6] Benbir G, Ince B, Bozluolcay M (2006). The epidemiology of post-stroke epilepsy according to stroke subtypes. Acta Neurol Scand.

[7] Lossius MI, Ronning OM, Slapo GD, Mowinckel P, Gjerstad L (2005). Poststroke epilepsy: occurrence and predictors--a long-term prospective controlled study (Akershus Stroke Study). Epilepsia.

[8] Camilo O, Goldstein LB (2004). Seizures and epilepsy after ischemic stroke. Stroke.

[9] Lamy C, Domigo V, Semah F, Arquizan C, Trystram D, Coste J, Mas JL, Patent Foramen O, Atrial Septal Aneurysm Study G (2003). Early and late seizures after cryptogenic ischemic stroke in young adults. Neurology.

[10] Cheung CM, Tsoi TH, Au-Yeung M, Tang AS (2003). Epileptic seizure after stroke in Chinese patients. J Neurol.

[11] Bladin CF, Alexandrov AV, Bellavance A, Bornstein N, Chambers B, Cote R, Lebrun L, Pirisi A, Norris JW (2000). Seizures after stroke: a prospective multicenter study. Arch Neurol.

[12] Arboix A, Garcia-Eroles L, Massons JB, Oliveres M, Comes E (1997). Predictive factors of early seizures after acute cerebrovascular disease. Stroke.

[13] So EL, Annegers JF, Hauser WA, O'Brien PC, Whisnant JP (1996). Population-based study of seizure disorders after cerebral infarction. Neurology.

[14] Hesdorffer DC, Benn EK, Cascino GD, Hauser WA (2009). Is a first acute symptomatic seizure epilepsy? Mortality and risk for recurrent seizure. Epilepsia.

[15] Sykes L, Wood E, Kwan J (2014). Antiepileptic drugs for the primary and secondary prevention of seizures after stroke. Cochrane Database Syst Rev.

[16] Gilad R (2012). Management of seizures following a stroke: what are the options?. Drugs Aging.

[17] Neshige S, Kuriyama M, Yoshimoto T, Takeshima S, Himeno T, Takamatsu K, Sato M, Ota S (2015). Seizures after intracerebral hemorrhage; risk factor, recurrence, efficacy of antiepileptic drug. J Neurol Sci.

[18] Morgenstern LB, Hemphill JC, Anderson C, Becker K, Broderick JP, Connolly ES, Greenberg SM, Huang JN, MacDonald RL, Messe SR (2010). Guidelines for the management of spontaneous intracerebral hemorrhage: a guideline for healthcare professionals from the American Heart Association/American Stroke Association. Stroke.

[19] Hong KS, Bang OY, Kang DW, Yu KH, Bae HJ, Lee JS, Heo JH, Kwon SU, Oh CW, Lee BC (2013). Stroke statistics in Korea: part I. Epidemiology and risk factors: a report from the korean stroke society and clinical research center for stroke. J Stroke.

[20] Beleza P (2012). Acute symptomatic seizures: a clinically oriented review. Neurologist.

[21] Marson A, Jacoby A, Johnson A, Kim L, Gamble C, Chadwick D (2005). Immediate versus deferred antiepileptic drug treatment for early epilepsy and single seizures: a randomised controlled trial. Lancet.

[22] Gilad R, Lampl Y, Eschel Y, Sadeh M (2001). Antiepileptic treatment in patients with early postischemic stroke seizures: a retrospective study. Cerebrovasc Dis.

[23] Karhunen H, Jolkkonen J, Sivenius J, Pitkanen A (2005). Epileptogenesis after experimental focal cerebral ischemia. Neurochem Res.

[24] Back T, Ginsberg MD, Dietrich WD, Watson BD (1996). Induction of spreading depression in the ischemic hemisphere following experimental middle cerebral artery occlusion: effect on infarct morphology. J Cereb Blood Flow Metab.

[25] Huang CW, Saposnik G, Fang J, Steven DA, Burneo JG (2014). Influence of seizures on stroke outcomes: a large multicenter study. Neurology.

[26] Burneo JG, Fang J, Saposnik G (2010). Investigators of the Registry of the Canadian Stroke N: Impact of seizures on morbidity and mortality after stroke: a Canadian multi-centre cohort study. Eur J Neurol.

[27] Berg AT, Berkovic SF, Brodie MJ, Buchhalter J, Cross JH, van Emde BW, Engel J, French J, Glauser TA, Mathern GW (2010). Revised terminology and concepts for organization of seizures and epilepsies: report of the ILAE Commission on Classification and Terminology, 2005–2009. Epilepsia.

[28] Fisher RS, Acevedo C, Arzimanoglou A, Bogacz A, Cross JH, Elger CE, Engel J, Forsgren L, French JA, Glynn M (2014). ILAE official report: a practical clinical definition of epilepsy. Epilepsia.

[29] Nair PP, Kalita J, Misra UK (2011). Status epilepticus: why, what, and how. J Postgrad Med.

[30] Beghi E, Carpio A, Forsgren L, Hesdorffer DC, Malmgren K, Sander JW, Tomson T, Hauser WA (2010). Recommendation for a definition of acute symptomatic seizure. Epilepsia.

[31] Adams HP, Bendixen BH, Kappelle LJ, Biller J, Love BB, Gordon DL, Marsh EE (1993). Classification of subtype of acute ischemic stroke. Definitions for use in a multicenter clinical trial. TOAST. Trial of Org 10172 in Acute Stroke Treatment. Stroke.

[32] Strzelczyk A, Haag A, Raupach H, Herrendorf G, Hamer HM, Rosenow F (2010). Prospective evaluation of a post-stroke epilepsy risk scale. J Neurol.

[33] Kim LG, Johnson TL, Marson AG, Chadwick DW (2006). Prediction of risk of seizure recurrence after a single seizure and early epilepsy: further results from the MESS trial. Lancet Neurol.

[34] Velioglu SK, Ozmenoglu M, Boz C, Alioglu Z (2001). Status epilepticus after stroke. Stroke.

[35] Fisher RS, van Emde BW, Blume W, Elger C, Genton P, Lee P, Engel J (2005). Epileptic seizures and epilepsy: definitions proposed by the International League Against Epilepsy (ILAE) and the International Bureau for Epilepsy (IBE). Epilepsia.

[36] Kim JH, Lee HW, Cohen LG, Park KD, Choi KG (2008). Motor cortical excitability in patients with poststroke epilepsy. Epilepsia.

[37] Petrides G, Braga RJ, Fink M, Mueller M, Knapp R, Husain M, Rummans T, Bailine S, Malur C, O'Connor K (2009). Seizure threshold in a large sample: implications for stimulus dosing strategies in bilateral electroconvulsive therapy: a report from CORE. J ECT.

[38] Stein DG (2008). Progesterone exerts neuroprotective effects after brain injury. Brain Res Rev.

[39] Kraus JA, Berlit P (1998). Cerebral embolism and epileptic seizures--the role of the embolic source. Acta Neurol Scand.

[40] Willmore LJ, Ueda Y (2009). Posttraumatic epilepsy: hemorrhage, free radicals and the molecular regulation of glutamate. Neurochem Res.

[41] Carrera E, Michel P, Despland PA, Maeder-Ingvar M, Ruffieux C, Debatisse D, Ghika J, Devuyst G, Bogousslavsky J (2006). Continuous assessment of electrical epileptic activity in acute stroke. Neurology.

