# Erratum to: clinical predictors of seizure recurrence after the first post-ischemic stroke seizure

**DOI:** 10.1186/s12883-017-0859-5

**Published:** 2017-05-04

**Authors:** Hyeon Jin Kim, Kee Duk Park, Kyoung-Gyu Choi, Hyang Woon Lee

**Affiliations:** Departments of Neurology, Ewha Womans University School of Medicine and Ewha Medical Research Institute, Epilepsy and Sleep Center, Ewha Womans University Mokdong Hospital, 1071, Anyangcheon-ro, Yangcheon-gu, Seoul, 158-710 South Korea

## Erratum

After publication of the original article [1], it came to the authors’ attention that there was an error in the abstract under the Methods heading. The first sentence reads as follows: “We reviewed 3792 ischemic stroke patients from the Ewha Stroke Registry”.

This sentence should be amended to the following: “We reviewed 3792 ischemic stroke patients who had admitted to Ewha Womans University hospital between 2001 and 2012.”

The ‘Ewha Stroke Registry’ is made from the medical records of our hospital. But the Registry is specifically selected & enrolled patient’s data for the statistical analysis.
